# Dental Chemistry and Metallurgy

**Published:** 1897-06

**Authors:** 


"Dental Chemistry and Metallurgy. By Clifford Mitchell, A. M., M. D. Fourth Edition. Publishers ; The W. T. Keener Company, Chicago.

The first edition of this work met with the approval of the dental profession, and proved a valuable aid to the student. Every succeeding edition has met with a still higher appreciation, and this new (the fifth) edition cannot fail to receive a more welcome reception even than any of the preceding ones. It is certainly abreast of the times, and the endeavor of its author to provide a course which shall include a large number of exercises in experimental inorganic and organic chemistry is manifested by the addition of more than one hundred new pages. The contents of this new edition also embrace physiological chemistry in which the process of digestion is considered at some length, thus affording a valuable addition to the treatise. The germ theory also receives greater attention, as well as new alkaloids and antiseptics, all of which add to the value of this treatise as a dental text book for the student.

